# Mechanism of Oxidation of 3:4-Benzpyrene in the Presence of Autoxidizing Thiols

**DOI:** 10.1038/bjc.1950.25

**Published:** 1950-06

**Authors:** G. Calcutt


### MECHANISM OF OXIDATION OF 3:4-BENZPYRENE IN THE PRESENCE OF AUTOXIDIZING THIOLS.

G. CALCUTT.

*From the Department of Cancer Research, Mount Vernon Hospital, and the Radium Institute, Northwood, Middlesex.*

Received for publication April 27, 1950.

EVIDENCE offered by Calcutt (1949) showed that 3:4-benzpyrene is effective in inducing the oxidation of the –SH groups of a variety of compounds, the benzpyrene itself also undergoing oxidation during this process. On the basis of physical properties and fluorescence spectra it was suggested that the benzpyrene derivatives obtained in this fashion were comparable to those obtained as a result of the metabolism of the hydrocarbon by mice. This suggestion is important in view of Crabtree's (1947) conclusions as to the involvement of –SH groups in the carcinogenic process, and also in relation to Powell and Calcutt's (1949) conclusion that the availability of sulphydryl influences the metabolism of benzpyrene. The present paper reports an attempt at the solution of the problem of the mechanism of the oxidative processes undergone by benzpyrene in the presence of autoxidizing thiols.

Studies by Weigert and Mottram (1946*a*, 1946*b*) showed that the initial step in the metabolism of benzpyrene is conversion to either 8:9-dihydro 8:$OR_1$ –9.OH benzpyrene ($BpX_1$) or 8:9-dihydro 8:$OR_1$ –9$OR_2$ benzpyrene ($BpX_2$), $R_1$ and $R_2$ being unidentified radicals. At the same time it was shown that these compounds were convertible by simple chemical methods, which have their counterpart in the animal body, to the final excretion products—8.OH benzpyrene and the 5:8 quinone. From the *in vitro* experiments with thiols Calcutt (1949) isolated a compound comparable to $BpX_1$ and in turn convertible, by the same methods used by Weigert and Mottram (1946*a*, 1946*b*), to a compound apparently identical with 8.OH benzpyrene. It is then with the formation of this $BpX_1$ type compound that the problem is concerned.

It was suggested by Quick (1937) that the primary step in the metabolic oxidation of hydrocarbons is a union of cysteine with the unsubstituted aromatic ring, replacement of the mercapturic acid by a hydroxyl group occurring later. There is no direct evidence for this view, but the recognized involvement between carcinogenesis and sulphur metabolism (Crabtree, 1947 ; Calcutt, 1949) is in its favour. It is, however, severely criticized by Boyland and Weigert (1947), who point out that phenylcysteine is excreted as phenylmercapturic acid—evidence which suggests that the linkage between cysteine and an aromatic compound is not easily broken in the body.

Consideration of the metabolism of dibenzanthracene led Fieser (1941) to suggest the addition of hydrogen peroxide as the primary metabolic process. Loss of water would then give the phenolic derivative. A similar perhydroxylation mechanism has been suggested by Porteous and Williams (1949) as the only feasible explanation of the isolated end-products of the metabolism of benzene. It is apparent that such a scheme could apply equally well in the case of the chemically related benzpyrene, $BpX_1$ or $BpX_2$ arising from the substitution of the hydrogen of one or other of the hydroxyl groups of the primarily formed dialcohol.

In the case of the *in vitro* experiments no evidence was found to indicate any linkage of the hydrocarbon and the thiol. Equally, even if such linkage had taken place it is difficult to conceive how the replacement by a hydroxyl group could have occurred under the simple conditions used. An alternative possibility in line with the perhydroxylation view is feasible since autoxidizing thiols are known to give rise to hydrogen peroxide (Schoberl, 1932; Holtz and Triem, 1937; Schales, 1938). Additionally, Weil-Malherbe (1947) claimed that benzpyrene was oxidized by hydrogen peroxide. Unfortunately no results are recorded as to the nature of the oxidized products.

If a perhydroxylation process is the initial step in both the *in vivo* and *in vitro* oxidation of benzpyrene it appears that it should be possible—under suitable conditions—to oxidize the hydrocarbon directly with hydrogen peroxide. This has been attempted, and the results are detailed below.

### METHODS.

*The oxidation of* 3:4-*benzpyrene with hydrogen peroxide.*

The addition of 6 per cent hydrogen peroxide to a colloidal suspension of benzpyrene in distilled water had no effect at room temperature even when the two compounds were maintained together for long periods. If, however, a similar mixture was maintained at 55°C. for 30 minutes the typical yellow-green fluorescence of the benzpyrene was replaced by a blue fluorescence of the type associated with benzpyrene derivatives. Continued warming led to the apparent complete disappearance of the benzpyrene and the formation of a brownish, insoluble flocculent material. Since hydrogen peroxide contains acid as a stabilizing agent, any derivative of the $BpX_1$ type formed in the reaction would be expected to break down to the phenol and thence to the quinone—a sequence of events which superficially would resemble those found.

As an attempt to counteract any influence due to the acid the experiment was repeated using the benzpyrene colloid suspended in a Clark and Lubbs buffer solution at a pH of 7·5. This time the hydrogen peroxide was added slowly; whilst at intervals the pH was checked, and when necessary was restored to 7·5 by the further addition of 0·2M. NaoH solution. After 30 minutes the reaction had reached the blue fluorescent stage so the mixture was cooled in the attempt to stop further action.

### RESULTS.

*The isolation and examination of the reaction products.*

The cooled reaction mixture was repeatedly extracted with purified (fluorescence free) xylene. The xylene was dried over anhydrous sodium sulphate and then passed through a chromatograph column packed with silica. After repeated washing with clean xylene there remained at the top of the column an extended zone which fluoresced pale blue under the U.V. lamp.

The column was extruded and the fluorescent zone was divided into three portions. All three were then extracted with methyl alcohol. Examination of the fluorescent spectra of the solution derived from the three portions gave results as below :

Surface :—Two diffuse maxima at 420 mμ. and 440 mμ. Indistinguishable from fluorescence spectrum of $BpX_1$ derived from mice.

Middle :—Maxima as above but overshadowed by a diffuse background.

Bottom :—One extended diffuse zone without banded structure.

Further examination of the material from the surface zone showed it to be water-soluble with a pale blue fluorescence. The addition of a few drops of acid converted this material to an alkali-soluble product having a fluorescence spectrum identical with that of 8.OH benzpyrene. The likelihood of it actually being 8.OH benzpyrene is enhanced by the fact that after transfer to petroleum ether and standing for a few days it lost its blue fluorescence and achieved a barely perceptible yellowish tinge, behaviour which would be in keeping with the known autoxidation of 8.OH benzpyrene to benzpyrene 5:8 quinone. The solution was therefore passed through a silica column, which resulted in the formation of a faintly tinted zone. The passage of a weak solution of an authentic sample of 3:4-benzpyrene 5:8-quinone through the same column resulted in concentration over the previous zone, and, although the zone slowly passed down the column, no further elution or variation in solvent allowed any separation of the two zones. This must be regarded as confirmatory evidence that the derived material was benzpyrene 5:8 quinone. The similarity of fluorescence spectra, physical properties and behaviour suggests then that the material separated from the reaction mixture is similar to, even if not identical with, the $BpX_1$ from mice and the $BpX_1$-like material formed in the presence of autoxidizing thiols.

Further examination of the substances extracted from the other two portions of the original chromatograph column produced little of interest other than that the material was water-soluble in both cases. Acid had no determinable effect. The very limited amounts of substance available severely hindered any detailed examination, and thereby preclude any conclusions as to the nature of these compounds. If, however, a benzpyrene diol was formed, as appears likely from the evidence above, it is possible that these latter compounds are further oxidation products. In view of Boyland and Levi's (1935) finding that dihydroxydihydroanthracene is very susceptible to oxidation, such an occurrence would seem most probable.

Examination of the eluate from the original chromatograph column showed the presence of unchanged benzpyrene only.

*Attempts to improve the reaction yield.*

Despite repeated experiments it has so far proved impossible to obtain more than very small amounts of the reaction products. The reason for this appears to be that in the presence of the buffer the benzpyrene colloid undergoes coagulation, with a subsequent reduction in surface area. As soon as the benzpyrene becomes coated with the blue fluorescing material, which is insoluble in the alkaline buffer medium, the reaction ceases. Attempts to utilize the benzpyrene in solution in organic solvents have so far failed, the only results being the formation of small amounts of apparent 8.OH benzpyrene and quantities of various unidentifiable products. Such systems undoubtedly introduce the further problem of the relative ease of oxidation of the solvent as compared with the benzpyrene. For the moment therefore the question of the isolation of sufficient of the $BpX_1$ type material for chemical identification is still unsolved.

### DISCUSSION.

The work described above indicates that 3:4-benzpyrene can engage in direct action with hydrogen peroxide. Furthermore, the evidence suggests that the primary reaction product is a diol closely resembling that found as the initial product of benzpyrene metabolism. At the same time this primary product shows a remarkable resemblance to that found as the result of oxidation of benzpyrene in the presence of autoxidizing thiols. In view of the known formation of hydrogen peroxide during the autoxidation of –SH groups, it seems probable that the primary mechanism of oxidation in both cases consists of the addition of hydrogen peroxide to the hydrocarbon. Equally, since the formation of peroxide is a phenomenon known to occur during biological oxidations (Oppenheimer and Stern, 1939), it seems that such a mechanism could account for the initial steps of the *in vivo* oxidation of benzpyrene.

At the moment, however, no specific conclusions can be drawn, since the identity of the three similar substances cannot be proved. The problem must await the discovery of conditions which will allow of the isolation of the oxidation products in such quantity as to allow complete characterization.

### SUMMARY.

The oxidation of 3:4-benzpyrene with hydrogen peroxide and the isolation of a product similar to the benzpyrene oxidation products obtained during metabolism and during oxidation in the presence of autoxidizing thiols is described. It is suggested that the oxidation of benzpyrene in the presence of autoxidizing thiols is due to hydrogen peroxide formation.

The expenses of this research were defrayed out of a block grant from the British Empire Cancer Campaign.

### REFERENCES.

Boyland, E., and Levi, A. A.—(1935) *Biochem. J.*, **29**, 2679.
*Idem* and Weigert, F.—(1947) *Brit. med. Bull.*, **4**, 354.
Calcutt, G.—(1949) *Brit. J. Cancer*, **3**, 306.
Crabtree, H. G.—(1947) *Brit. med. Bull.*, **4**, 345.
Fieser, L. F.—(1941) " Production of Cancer by Polynuclear Hydrocarbons." University of Pennsylvania Bicentennial Conference.
Holtz, P., and Triem, G.—(1937) *Z. physiol. Chem.*, **248**, 1.
Oppenheimer, C., and Stern, K. G.—(1939) ' Biological Oxidation.' The Hague (W. Junk).
Porteous, J. W., and Williams, R. T.—(1949) *Biochem. J.*, **44**, 56.
Powell, A. K., and Calcutt, G.—(1949) *Brit. J. Cancer*, **3**, 430.
Quick, A. J.—(1937) *Ann. Rev. Biochem.*, **6**, 299.
Schales, O.—(1938) *Ber. chem. Ges. Frankfurt*, **71b**, 447.
Schoberl, A.—(1932) *Z. physiol. Chem.*, **209**, 231.
Weigert, F., and Mottram, J. C.—(1946*a*) *Cancer Res.*, **6**, 97.—(1946*b*) *Ibid.*, **6**, 109.
Weil-Malherbe, H.—(1947) *Brit. J. Cancer*, **1**, 410.


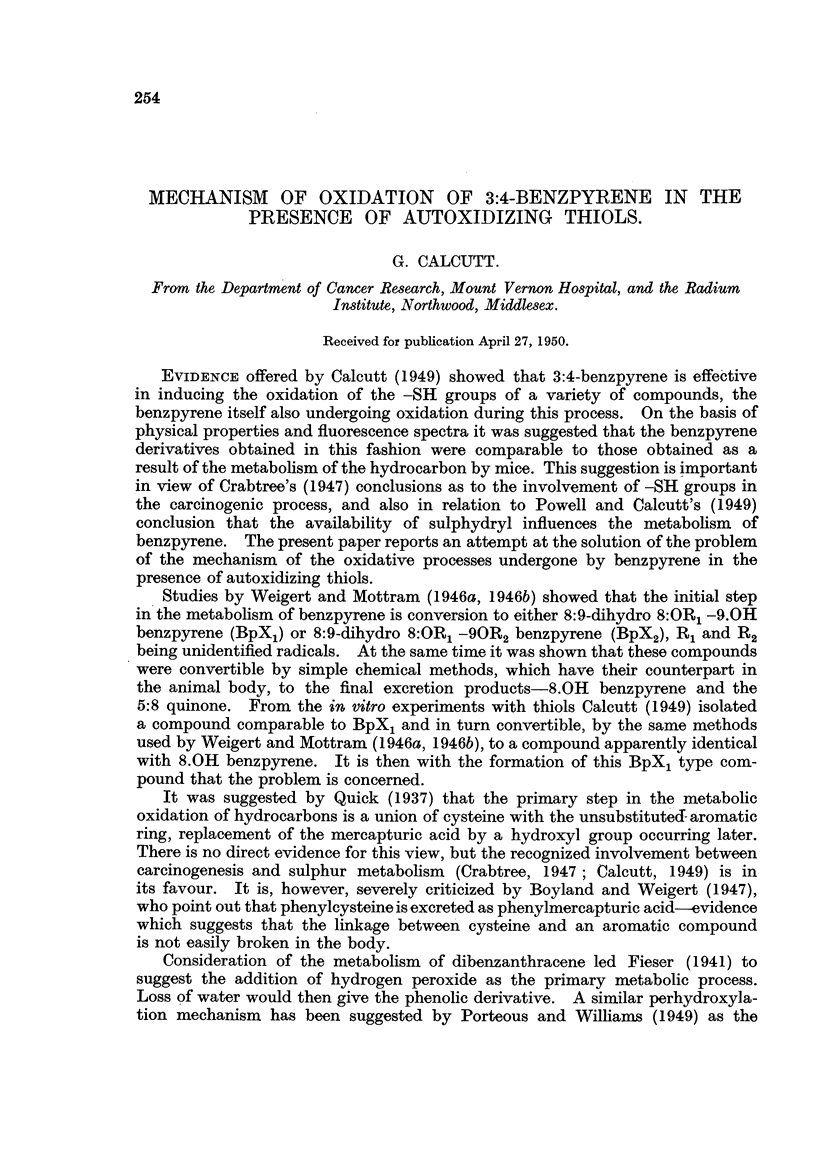

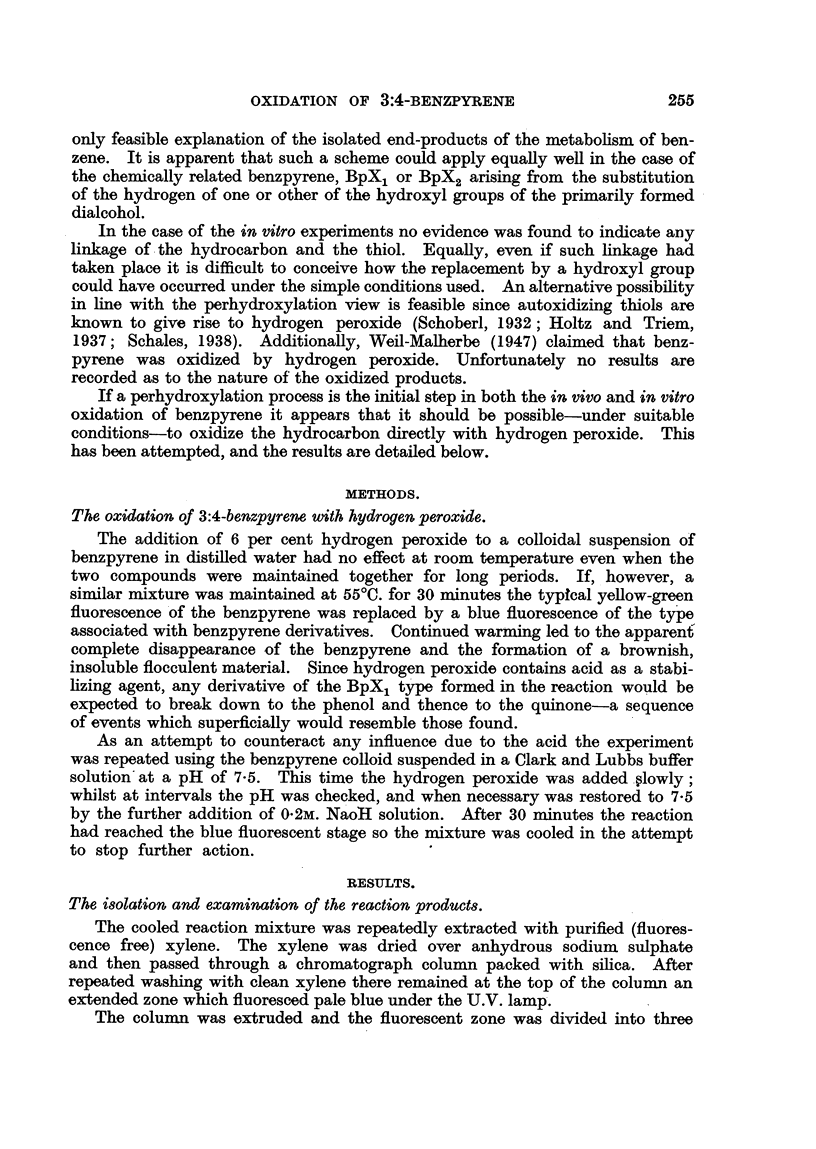

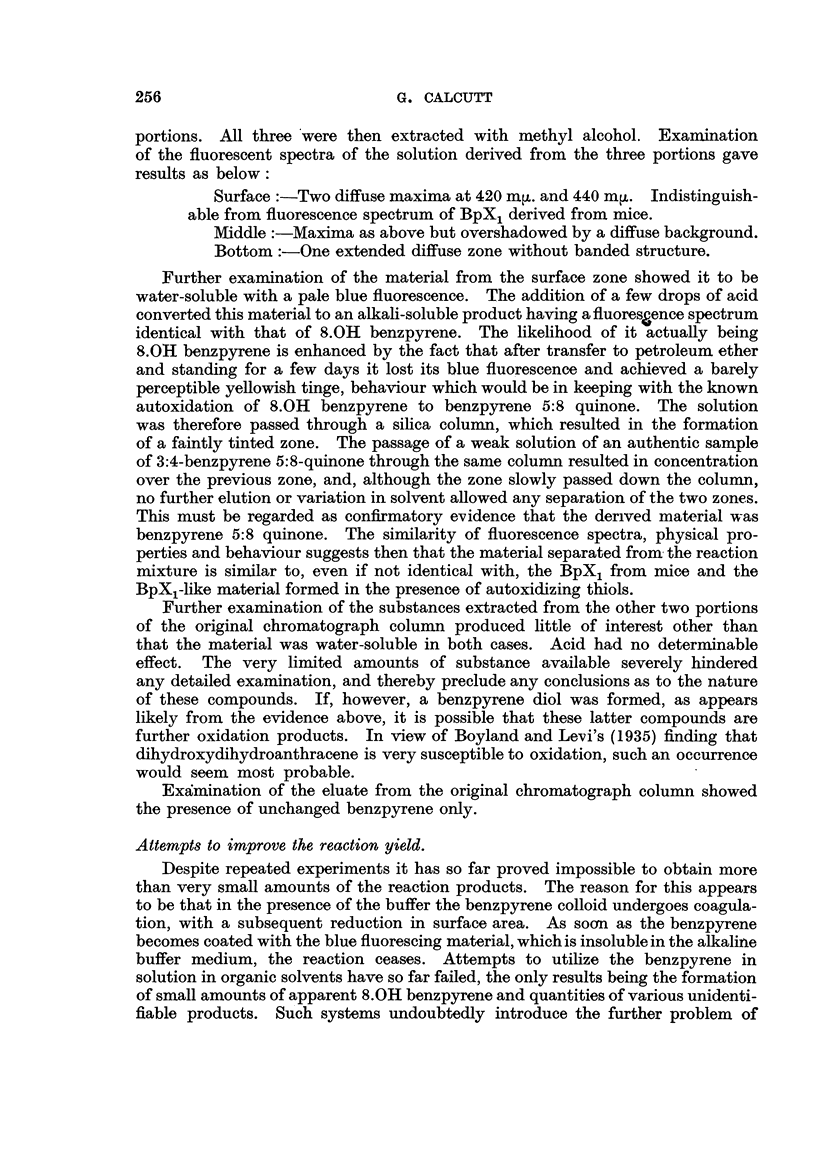

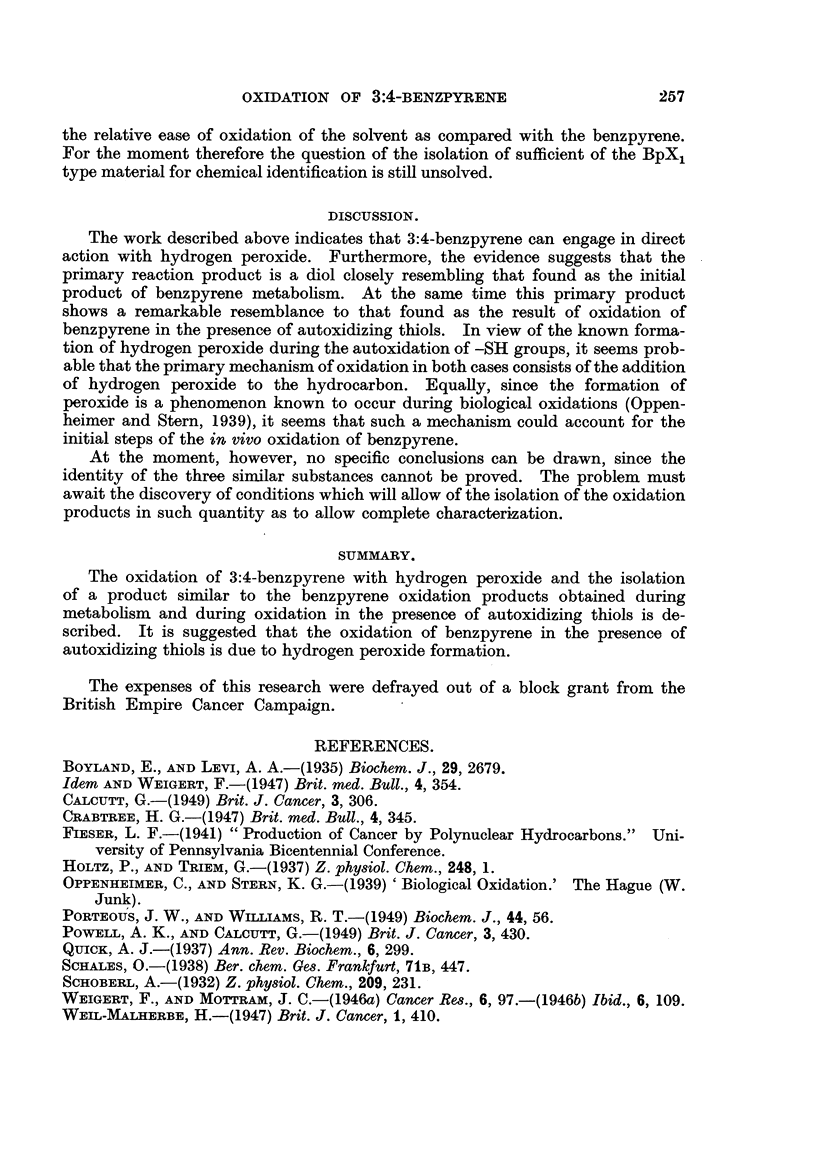


## References

[OCR_00216] Boyland E., Levi A. A. (1935). Metabolism of polycyclic compounds: Production of dihydroxydihydroanthracene from anthracene.. Biochem J.

[OCR_00233] POWELL A. K., CALCUTT G. (1949). Further experiments on the in vitro metabolism of 3:4-benzpyrene in the mouse skin.. Br J Cancer.

